# Scratch lottery tickets are a poor incentive to respond to mailed questionnaires

**DOI:** 10.1186/1471-2288-6-19

**Published:** 2006-04-28

**Authors:** Vilhjalmur Finsen, Andreas H Storeheier

**Affiliations:** 1 Dept. of Orthopaedic Surgery, St. Olav's University Hospital, 7006 Trondheim, Norway; 2 Dept. of Neuroscience, Faculty of Medicine, Norwegian University of Science and Technology, 7005 Trondheim, Norway

## Abstract

**Background:**

It has been demonstrated that the enclosure of money with a mailed questionnaire increases the response rate significantly. We evaluated scratch lottery tickets as an alternative to cash.

**Methods:**

1500 randomly selected Norwegians between the ages of 40 and 65 years were sent a short questionnaire. 250 received one lottery scratch ticket worth 20 Norwegian kroner (approximately 3 US$) together with the questionnaire, 250 received two scratch tickets, and 250 were promised two scratch tickets if they replied within one week. A fourth group of 250 persons received a 50 kroner banknote with the questionnaire. The remaining 500 letters served as controls.

**Results:**

The overall response rate after 6 weeks was 77%. Logistic regression analysis showed that only the 50 kroner group had a response rate that was statistically significantly higher than the controls (p < 0.0001). It was also significantly higher than that in any of the other incentive groups (p < 0.0001, p < 0.004 and p < 0.0001 respectively). Female sex (p < 0.001) and age (p < 0.002) increased the response rate significantly.

**Conclusion:**

It is possible that the recipients scratched their cards before completing the questionnaire, and that it was a disincentive for the majority that they did not win anything. Lottery scratch tickets are no substitute for cash as an incentive to respond to a questionnaire.

## Background

A great effort is usually made to induce as many as possible to reply in any study employing questionnaires. Many of the factors that influence the response rate, such as the interest to the recipient of the subject under investigation and the length of the questionnaire, can not easily be altered [1,2]. It has been shown that the response rate may be increased by incentives like using impressive postage stamps [3] offering participation in a lottery [4,5] and particularly by enclosing money [1,6-12]. However, sending cash in the mail is frowned upon by the postal authorities in many countries and some recipients might be offended by being offered money to participate in a scientific investigation.

The purpose of this study was to test if scratch lottery tickets used in various ways might serve as a substitute for cash.

## Methods

A list of 1500 randomly selected residents of Norway between the ages of 40 and 65 was supplied by the Norwegian Census Bureau. Each was sent a questionnaire asking if any of a list of specified operations had been performed on the recipient or his immediate family. Enclosed were also a stamped return envelope and a letter explaining that the purpose of the survey was to determine if these operations were performed more or less frequently in the general population than among physicians and their families as this might indicate that the operations were performed too often or too rarely in the general population [13]. In a pilot study 10 recipients spent 4 (range 2.5 – 5) minutes completing the form.

Before mailing, recipients were randomized by rearranging them in alphabetical order according to the first name of each person. The first 250 received one scratch ticket for a lottery conducted by the Norwegian Society for the Blind, the second 250 received two such scratch tickets, and the third 250 were promised two scratch tickets if they replied within one week. Each lottery scratch ticket clearly displays the retail price of 20 Norwegian kroner (NOK), approximately equal to 3 US$. An area of the ticket is covered by a film that can be scratched away to reveal nine numbers. If three of the numbers are the same, the owner wins the corresponding sum in NOK.

The next 250 persons on the list received a 50 NOK note with the initial letter. The remaining 500 letters were sent without any particular incentive and served as controls.

All the enclosed letters were the same except that those sent to others than the controls contained a final brief paragraph explaining that the reward was a token of gratitude for their taking part in the study.

A reminder, including a new form and stamped reply envelope, was sent to those who had not replied after three weeks. The number of replies was counted at the end of each week for the first six weeks after the initial mailing.

Permission for the study was given by the Regional medical research ethics committee. The chi square test was used for comparison of proportions. The response rates were fitted in a logistic regression model with age, sex and incentive as covariates. P-values of less than 0.05 were taken to indicate significant differences.

## Results

Randomization had lead to a very similar mean age in the groups, but there were fewer women than average in the control group and more in the group that received one scratch ticket (Table 1).

**Table 1:** Age- and sex-distribution of the groups

	**Mean age**	**Women**	**Men**	**% Women**
1 ticket	51.7	140	110	56.0
2 tickets	51.2	133	117	53.2
2 tickets later	51.5	123	127	49.2
50 NOK	51.0	126	124	50.4
Controls	51.4	197	303	39.4
All groups	51.4	719	781	47.9

**Figure 1:**
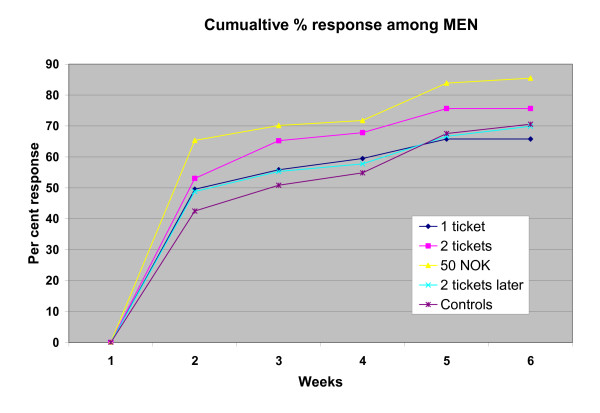
Cumulative response rate in the various groups among men at the end of each of the first 6 weeks.

**Figure 2:**
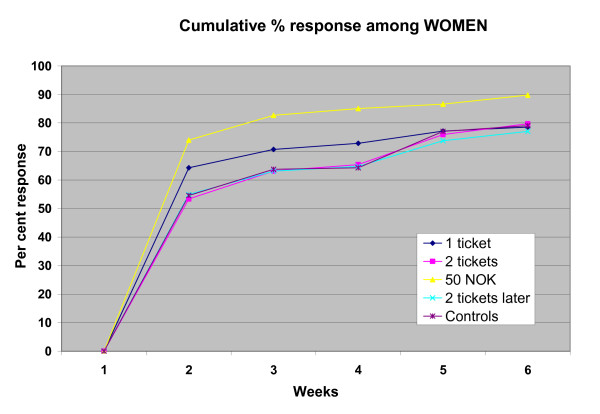
Cumulative response rate in the various groups among women at the end of each of the first 6 weeks.

14 questionnaires were returned by the post office because the recipients could not be traced and two recipients returned an uncompleted form together with the 50 NOK notes they had received. The first replies were received four days after the initial mailing. The cumulative number of replies during the first 6 weeks is shown separately for women and men in figures 1 and 2.

The overall response rate after 6 weeks was 77 percent. It was 73 percent among those who had been sent one scratch ticket and also among those who had been promised 2 tickets if they answered within a week, 74 percent among the controls, 78 percent among those who had received two tickets, and 88 percent among those who had received 50 NOK.

Logistic regression analysis of responses after 6 weeks revealed that when results were corrected for age and sex differences in the groups, only the response rate from those who had received 50 NOK was significantly different from that of the controls (Table 2) and that the response rate was significantly higher in this group than in any of the other incentive groups (one ticket: p < 0.0001; two tickets: p < 0.004; two tickets later: p < 0.0001). No statistically significant interactions were present between sex, incentive and age.

**Table 2:** Results of logistic regression analysis of responses in the various incentive groups compared to the controls after 6 weeks and of the effects of sex and age.

	**Odds ratio**	**95% conf. int.**	**P-value**
Controls (reference group)	1		
1 ticket	0.881	0.620–1.250	0.5
2 tickets	1.209	0.836–1.748	0.3
2 tickets later	0.920	0.647–1.309	0.6
50 NOK	2.489	1.612–3.841	0.0001
Female sex	1.547	1.204–1.987	0.001
Age (per year)	1.028	1.010–1.046	0.002

The overall response rate from women was 81 percent and that from men 73 percent (p < 0.0007). Logistic regression showed that the higher response rate persisted after correcting for age and incentive group (Table 2).

Overall, the older half of the recipients, those older than 52 years, had a reply rate of 79 percent which was slightly higher then the 74 percent of their younger counterparts (p < 0.02). Logistic regression showed a statistically significantly increased response rate with age also when sex and incentive group were taken into account (Table 2).

## Discussion

A review of 18 papers published between 1931 and 1973 studying monetary incentives in mail surveys reported that all showed that enclosing money with the questionnaire significantly increased the response rate [6]. On average the number of non-responders was reduced by one third. A recent thorough review which included 292 studies of which 49 compared monetary incentives to no incentive concluded that cash is the strongest incentive of all those that have been investigated, more than doubling the odds of response [1]. Increasing the amount of money leads to a higher response rate, but the marginal benefit tapers off rapidly with increasing sums.

Sending money only with the reminder is also effective, but less so than when the reward is enclosed in the first letter [6,8]. One intriguing observation is that the effect is greater of enclosing money in the first letter than promising it to those who reply, even when the promised reward is greater than that enclosed in the original mailing [6,8,9]. The special value of cash as an incentive is further demonstrated by the observation of a greater response rate with cash than with a cheque worth twice the amount [11].

It may have been unfortunate that we only promised two scratch tickets to those who responded within a week. Some may have delayed for various involuntary reasons for some days and the promise would then no longer have served as an incentive. Our study confirmed that the response rate is higher among older than younger recipients and among women than men, particularly when no or little reward is involved [12].

We enclosed the smallest Norwegian bank note in the letters to our cash group. Its value is slightly higher than the value of the two lottery scratch tickets received by the group with the second highest response rate, but it seems unlikely that this accounts for the great difference in the number of replies. It seems possible that many of the recipients of scratch tickets scratched their tickets before completing the questionnaire and that the tickets were no longer perceived as a reward by the majority who had not received a winning ticket. Our study confirms that very few recipients are offended by being sent cash, at least not to the extent that they return the money [7,9].

## Conclusion

Almost all studies of cash incentives to improve the response rate to questionnaires have been conducted in America. It seems that the findings in these studies also hold true for Northern Europe. Cash greatly reduces the rate of non-responders also in Norway. We further conclude that this effect can not be obtained by using lottery scratch tickets instead of money.

## Competing interests

The author(s) declare that they have no competing interests.

## Authors' contributions

VF conceived of and designed the study, analyzed the data, and wrote the manuscript. AHS participated in the design of the study, collected the data and read and approved the manuscript.

## Pre-publication history

The pre-publication history for this paper can be accessed here:


